# Urinalysis

**Published:** 1896-01

**Authors:** 


Urinalysis, including five hundred blanks, for recording the analysis and microscopic examination of the urine. For medical practitioners, specialists, life insurance companies, and pharmacists. Arranged by Joseph C. Guernsey, A. M., M. D. Large octavo. Cloth, $3.00. Extra blanks in pads of fifty, 50 cents. For sale by all booksellers, or will be sent by the publishers, free of expense, on receipt of the price. J. B. Lippincott Company, 715 and 717 Market Street, Philadelphia.

This work contains, in a few pages, the essentials of urinalysis, including the apparatus and chemical reagents needed; the chemical tests for albumen, bile, blood, chlorides, indican, phosphates, pus, solids, specific gravity, sugar, sulphates, urates, urea, uric acid, and urobilin; blanks for five hundred examinations.

The system of blanks used has been found by the author very convenient for rapidly recording in permanent form as much or as little of a urinary examination as may be desired.

The chief advantage of a book of this kind is that one can keep a continuous record, always at hand, of repeated urinary examinations. It is, therefore, of great use in showing the progress made in Bright's disease, diabetes, and all (other) renal disorders.

Nothing like this book has ever been seen by the editor. It may be more plainly described by saying that it is a collection of five hundred blanks for recording the state of the urine in every case of disease coming under the practitioner's notice, and that it is further furnished with descriptions and directions for testing urine, apparatus, and so on. It also has an index to these blanks where the name of the patient on the blank is entered. The blanks being numbered, it is easy to find any particular record.

It is a most unique book; one that should be in the hands of every physician, and carefully used by him in keeping the record of his critical cases.

